# Point of care antimicrobial susceptibility testing

**DOI:** 10.1007/s10096-026-05453-0

**Published:** 2026-03-17

**Authors:** Jora Couwenberg, Alex van Belkum, Daan van de Kerkhof, Volkher Scharnhorst, Heiman Wertheim, Ardjan van der Linden, Suzanne van Asten

**Affiliations:** 1 Department of Biomedical Engineering, Eindhoven University of Technology, Eindhoven, The Netherlands; 2 Department of Clinical Laboratory, Catharina Hospital, Eindhoven, The Netherlands; 3 ShanX Medtech, Eindhoven, The Netherlands; 4 Department of Medical Microbiology and Infectious Diseases, Radboud University Medical Center, Nijmegen, The Netherlands; 5 Independent Microbiology Advisor, Rijnsburg, The Netherlands; 6 Eindhoven University of Technology and Clinical Laboratory Catharina Hospital Eindhoven, De Rondom 70, Eindhoven, 5612 AP The Netherlands

**Keywords:** Point of care testing, Antibiotic susceptibility, Antibiotic resistance, Antimicrobial stewardship

## Abstract

Given the rising threat of antimicrobial resistance (AMR), there is an urgent clinical need for faster, near-patient antibiotic susceptibility testing (AST) solutions. Standard AST methods usually need pure bacterial isolates, with results available in at minimum 18–24 h, leading to a total turnaround time (TAT) of 1–3 days. Financial pressures from healthcare insurers, technological advancements, and the involvement of private investors have changed the laboratory landscape. This trend, together with the ongoing centralization of microbiological laboratories, has led to longer diagnostic TATs. The development and use of point-of-care testing (POCT) devices capable of delivering diagnostic and AMR results have the potential to improve patient outcomes, decrease hospital stay, lower healthcare costs, and improve antimicrobial stewardship. However, widespread adoption remains challenging due to technical complexities, limitations in pathogen detection, high costs, and regulatory barriers. The aim of this review is to outline the current state of affairs in AST focusing on POCT devices from the pre-clinical stage to commercial launching.

## Introduction

One of the leading public and nosocomial health threats of the 21st century is bacterial antimicrobial resistance (AMR) [1, 2]. The current rise in AMR is expected to kill 10 million people annually by 2050 [3, 4]. Healthcare expenses will be raised by the ongoing switch from generally cheaper first-line antibiotics to more costly treatments using the newer antibiotics, along with prolonged periods of illness and medical care. According to the World Bank, infections caused by resistant bacteria could trigger a global economic crisis, potentially pushing 28 million people into extreme poverty by 2050 [5]. The World Health Assembly endorsed a global action plan centered around five key strategic goals, including enhancing the responsible use of antimicrobial agents and advancing investment in innovative microbiological diagnostic technologies [6].

Rapid detection and identification of an infectious pathogen as well as fast determination of its antimicrobial susceptibility profile are essential for effective treatment of bacterial infections [7]. Antimicrobial susceptibility testing (AST) is a foundational component of diagnostic stewardship and is essential for AMR surveillance [8].

Classically, diagnosis of a bacterial infection is based on microbial cultivation, in which viable bacterial cells are amplified into large numbers [7]. These culture-based methods are labor-intensive, time-consuming and require highly-trained personnel [6]. The cultivated colonies can be immediately used for species identification using classical biochemistry and/or genomic technologies [9]. The same technologies and more can be applied to AST, depending on the technical format used.

Traditional AST techniques such as broth macro- or micro-dilution (BMD), disk diffusion, and gradient diffusion provide the current golden standard methods and yield reliable quantitative results. These phenotypic techniques assess observable characteristics of bacterial cells that arise from the interaction between their genetic and phenotypic make-up. Despite their widespread use, these methods offer limited experimental flexibility and are associated with several conceptual limitations. These include the need for visual and non-automated reading, all-manual procedures, and challenges such as the inhomogeneous growth of bacterial species resulting in long turnaround times (TATs), variability and complexity [7, 10]. In addition, these methods require regular re-calibration depending on local changes in the resistance distribution. The shortcomings of traditional AST are increasingly visible with the contemporary raise in AMR.

**Fig. 1:**
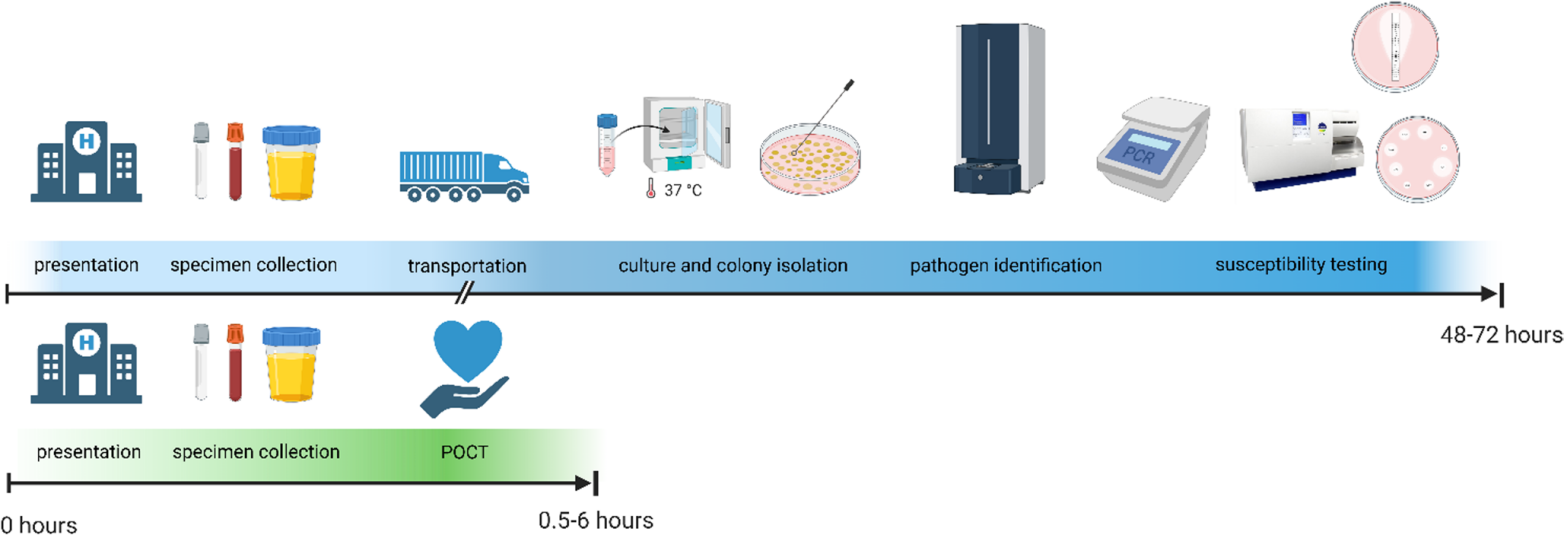
Schematic overview of turnaround times (TATs) from patient presentation and sample collection to results. Above, classical workflow and below the workflow after implantation of a point-of-care testing (POCT) device

Automated AST methods have been developed and are well-accepted in microbiology laboratories [11, 12]. Critical literature reviews are plentiful in number [5, 7, 13–15]. The market is dominated by a limited number of manufacturers and the most important downside of all these methods is the requirement of a pre-cultivation step to obtain bacterial isolates, leading to a TAT of 1–3 days at least (Fig. 1) [5, 15].

Building on the limitations of current methods, automation has driven centralization among microbiology laboratories due to the large physical footprint and high costs associated with automated equipment. This trend is expected to continue, even though it may lead to further delays in TAT resulting from the longer distances required to transport samples [16, 17].

The development and implementation of rapid diagnostic tests that can simultaneously identify bacterial infections and provide AST results without the need for isolated cultures, could significantly reduce TAT, enhance clinical decision-making, and enable timely antibiotic therapy [2, 3, 15]. Moreover, the development of small-footprint and near bedside diagnostic tools, also known as point-of-care testing (POCT) devices could further reduce the TAT (Fig. 1) [16, 18].

Besides the positive outcomes for individual patients that a rapid diagnosis provides, POCT is also considered to be a priority tool in combating the global issue of AMR [3]. Both the review on Antimicrobial Resistance by O’Neill and the latest Lancet series emphasize that diagnostic technologies should be improved [19, 20]. The European Health and Digital Executive Agency (HaDEA) has nominated the development of rapid POC AST devices as one of the Prior Information Notices under the EU4Health program [21, 22]. The Foundation for Innovative New Diagnostics (FIND) is working to support access to diagnostics globally, especially in low- and middle-income countries, again with a focus on affordable POCT devices [23].

Despite the promise of POCT devices and efforts by several organizations to improve global access, a significant gap persists between the clinical need for rapid diagnostics and their actual development and implementation [6, 24]. The purpose of this review is to define the state of affairs in AST focusing on POCT devices ranging from a pre-clinical phase to commercial launching. We aim to define the clinical benefits of different POC AST techniques in the developing laboratory landscape.

## POCT and AST: concept and challenges

During the past decades, POCT has played a significant role in the evolution of the healthcare industry [2]. Examples include blood-glucose monitoring tests, rapid antigen tests for COVID-19 and bedside C-reactive protein and procalcitonin tests [25]. POCT has the potential to improve and accelerate the diagnosis of bacterial infections [26] allowing for rapid initiation of appropriate therapy while avoiding antibiotic overuse and misuse [2, 24].

Most importantly, POCT devices must have a direct impact on patient care or aid in public health goals. Ideally, these tests are performed directly on the clinical specimen and meet the REASSURED criteria set by the WHO [18]. In practice, meeting all these criteria is challenging, and trade-offs are frequently necessary. However, the nature of these trade-offs can vary depending on the stakeholder, as different groups—such as clinicians, policymakers, manufacturers, or patients—may prioritize different criteria (e.g., affordability, sensitivity, or ease of use) [18]. In general, many geographic, logistic, technical and regulatory challenges must be overcome between the proof-of-concept and commercialization phases of a test with an acceptable accuracy, costs and complexity [15, 27].

For AST systems specifically, one of the greatest technical challenges is achieving standardized minimal inhibitory concentration (MIC) measurements directly from specimens. This is primarily due to the unknown bacterial concentrations and the ill-defined presence of AST inhibiting compounds [15, 27]. Accurate MIC determination requires an adequate range of antibiotic dilutions and must adhere to performance criteria established by the European Committee of Antimicrobial Susceptibility Testing (EUCAST) and/or International Organization for Standardization (ISO) [15, 28]. Moreover, MIC values must be translated into categorical interpretations using susceptibility breakpoints defined for individual species-antibiotic combinations [5, 29], which means that direct-from-specimen POCT may need to be combined with (partial) pathogen identification.

In addition, testing and optimizing AST systems for a relevant spectrum of antimicrobials is complex and demands well-considered design choices. This process is further influenced by the intended user setting of the POCT device, and requires extensive research that can be time-consuming and may not always yield the desired outcomes [15, 27].

## Commercially available POC AST

Currently, only one regulatory approved phenotypic POC AST device is commercially available, namely the PA-100 AST system (PA-100) developed by Sysmex Astrego (SYSMEX Europe SE) [15]. The PA-100 is validated for use in uncomplicated urinary tract infections (UTIs). The urine matrix is relatively free of proteins, cells and other potential inhibitory substances, with bacterial concentrations of > 10^5^ CFU/mL constituting a positive UTI. Therefore, it is a close-to-ideal matrix for direct-from-specimen testing [15, 30].

The PA-100 system integrates microfluidics with advanced microscopy to enable single-cell imaging. A microfluidic chip separates and isolates individual bacterial cells while filtering out larger components. Within this highly controlled environment, bacteria are incubated in the presence or absence of specific antibiotics. In addition to the direct observation of cell division, the system also measures the impact of antibiotic exposure by tracking the elongation of individual bacterial cells, providing a rapid assessment of growth dynamics. Software performs presumptive species identification to provide an antibiogram calibrated against the EUCAST Clinical Breakpoint tables. The system detects the presence of bacteriuria above 50,000 CFU/mL and provides AST results for five antibiotics commonly used to treat uncomplicated UTIs within 45 min. The instructions for use (IFU) states validation and thus usage of the system for the most frequent species causing uncomplicated UTIs: *Escherichia coli*, *Klebsiella pneumoniae*, *Proteus mirabilis*, *Enterococcus faecalis* and *Staphylococcus saprophyticus* [6, 31, 32].

A first independent clinical evaluation by *Alonso-Tarrés et al.*. compared the PA-100 with routine microbiology for the detection of UTIs and evaluated the AST performance using 278 urines from female patients. A sensitivity of 84.0% and specificity of 99.4% was found for detecting bacteriuria under IFU specifications. Outside the IFU specification, the sensitivity decreased to 65.7% while the specificity remained high at 99.3%. The overall categorical agreement for the five antimicrobials used to compare the PA-100 under IFU specification to routine practice (amoxicillin/clavulanic acid, ciprofloxacin, fosfomycin, nitrofurantoin and trimethoprim; disc diffusion and BMD), was found to be 94.6%. Critically, the NPV for resistance in the cohort was above 93% for all antibiotics tested, indicating that a susceptible test result could reliably be used for treatment decisions. Hypothetically, in this study, the frequency of optimally treated patients could have improved from 58.3% to 78.4% [6]. Another study presented an economic analysis to evaluate the impact of adopting the PA-100 across various settings in Spain. The model projected an average saving of EUR 119 per patient. However, the reliability of this figure is limited by the numerous assumptions and simplifications used in constructing the model [33].

Limitations of the PA-100 lie in the small panel of antibiotics and species, restricting the use to uncomplicated UTIs. The system does not disclose which microorganisms are assumed to be present in the culture, so there is no ability to immediately reflect on the breakpoints provided [6]. To enable the use of the PA-100 system for other clinical specimens such as blood or cerebrospinal fluid, the detection limit should be improved.

## POC AST systems close to commercial launching

Six other companies were identified working on POC AST devices as summarized in Table 1, but their systems have not yet passed regulatory approval [34–40]. They have revealed little about their technology and do not share any clinical testing data. The number of companies currently progressing toward regulatory approval remains limited, underscoring the inherent challenges associated with obtaining authorization. In a study conducted by *Arienzo et al.*, an POC AST device, was evaluated for the detection of uropathogens [41]. This system uses a colorimetric method that allows for semi-quantitative estimation of viable bacterial concentrations through the measurement of enzymatic activity. Although promising results were obtained, it was subsequently found that the company responsible for developing the device is no longer operational [41].

**Table 1:** Overview of companies developing a point of care testing device for antibiotic susceptibility testing close to commercial launching

Company	Platform Name	Platform Technology	Detection method	Claimed time to result	Claimed/ Desired matrix	Comments
Astek Diagnostics [34]	JIDDU™	Microfluidics with separation and analyzing chamber. Bacteria are captured and cultivated.	Monitors metabolic activity using fluorescent dye.	Diagnosis and AST within 1 h	• Urine • Cerebral spinal fluid • Effluent • Blood	Market launch expected in first quartile of 2026 for UTIs.
InnotiveDx [35]	InnotiveDx	Capturing bacteria in monolayer of microfluidic cartridge. Allow bacteria to grow rapidly.	Enumeration of bacteria, morphology determined and fluorescent dyes used to provide spectral fingerprint.	Diagnosis 10–60 min AST 60 min	• Urine	Now integrating their assay and gearing up. Also includes identification function.
Accelerate Diagnostics [36]	Accelerate Pheno^®^	Capturing of living cells on cassette surface. Allow growth and track phenotypic features.	Fluorescent in-situ hybridization (FISH).	Identification in 2 h AST in 7 h	Not specifically mentioned	Multiplexing capabilities. MIC values provided. Identification function.
ShanX Medtech [37]	-	Microfluidic cartridge with small growth chambers.	Monitors metabolic activity using fluorescent dye.	Diagnosis and AST 1 h	• Urine • Cerebral spinal fluid • Wound fluid • Blood	In Advanced clinical stage for UTIs. Expected identification function.
Pattern Bioscience [38]	Pattern	Capture living cells in single picovolume-scale droplets. Combine machine learning and single cell imaging to obtain single cell resolution.	Monitors metabolic activity over time using fluorescence.	AST and identification 5–8 h	All different types of samples	Provides rapid AST and identification results in all types of samples
First Light Diagnostics [39, 40]	MultiPath^®^ Platform	Perform digital imaging by combining fluorescently labeled DNA probes and fluorescent magnetic beads to capture the bacteria.	A digital camera detects the individual cells captured via the magnetic beads.	Diagnosis in 30 min AST in 4 h	Not specifically mentioned	AST is performed separately in another cartridge. Identification function. Some pre-clinical data for UTIs.

## Proof-of-concept approaches

**Fig. 2:**
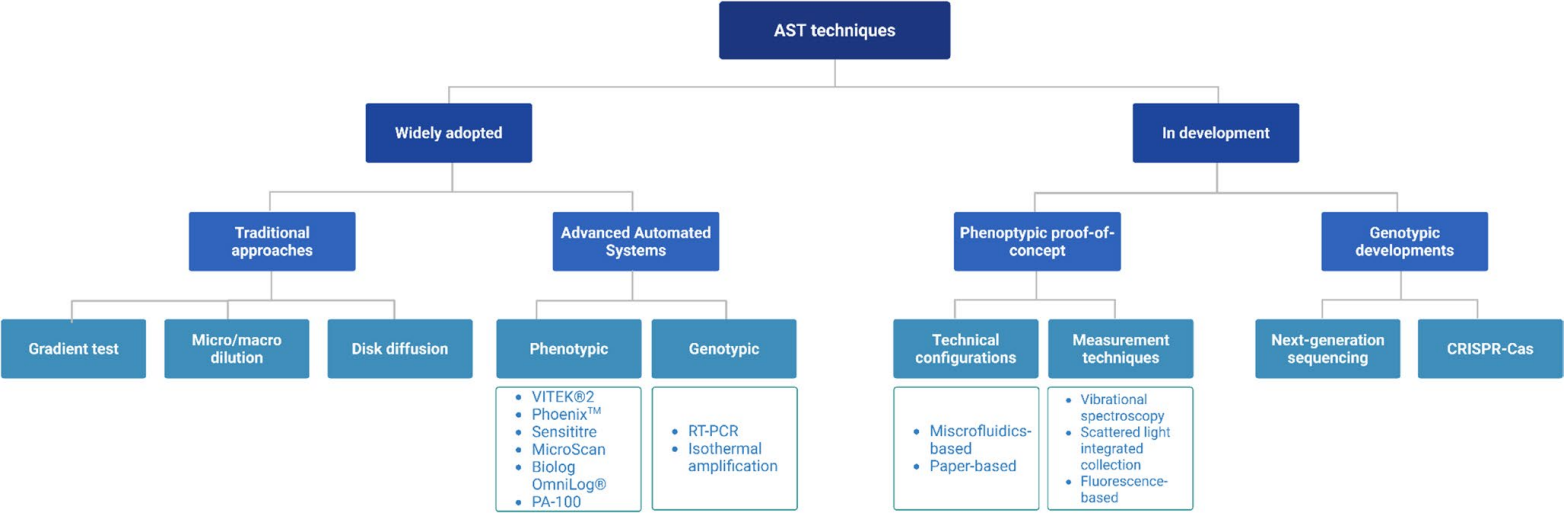
Overview of antibiotic susceptibility testing techniques discussed in this narrative review focusing on point of care antibiotic susceptibility testing for bacterial infections

Several innovative phenotypic methods for POC AST are currently under development (Fig. 2). These methods can be categorized into measurement techniques and technical configurations. The technical configurations primarily consist of microfluidic platforms and paper-based assays, both of which can be coupled with various photonic readout techniques to enhance analytical performance and adaptability. Photonic readout techniques include vibrational spectroscopy and fluorescence-based methods utilizing fluorescent or colorimetric dyes for pathogen detection [4].

### Miniaturized and low‑cost assay platforms

The basic technical configuration of clinical microbiology has always been culture based, using artificial liquid and semi-solid growth media. Such media have been refined over the past decades by better balancing the presence of nutrients, adding suppressive agents to allow for species specificity and completing with chromogenic compounds to allow direct species identification. Culture-based testing is macroscopic and there is a need for rendering this smaller in scale. An interesting development is the use of miniaturized dipstick containing many compartments filled with chromogenic agar. This allows for the quantification of bacteria in suspension, simultaneous with their chromogenic quantification [26].

#### Microfluidics-based

technologies find applications in environmental surveillance, food security, and healthcare diagnostics [42–44]. Microfluidics encompasses the study and application of fluid dynamics, heat transfer, and mass transport at the micrometer scale. In the field of AST, the use of microfluidics is often seen to obtain a rapid output, precise fluid control, and utilization of minimal amounts of samples/reagents. On top of that, it can provide automation, mobility and process integration of the platform into a POCT device leading to minimized human interventions as well as much lower error rates [14, 44, 45]. Microfluidic devices can easily be combined with a wide variety of electrical, magnetic, and optical characterization methods, allowing for fast identification and detection in just minutes [44]. Microfluidic technologies for antibacterial resistance study and AST have been reviewed [44, 46] and an overview of the challenges encountered for integration of microfluidics in a POCT were evaluated [27]. While microfluidic approaches for AST have shown promising proof‑of‑concept success in research settings, their translation into clinically impactful tools for combating antimicrobial resistance remains slow and dependent on sustained, coordinated collaboration among stakeholders. Emerging capabilities in smartphones, sensing, microscopy, and paper microfluidics enable simplified phenotypic AST, but clinical sample complexity remains a key challenge for widespread implementation.

#### Paper based

devices are easy-to-use, cost-effective and portable. They are often combined with a colorimetric readout approach. However, *Rafiee et al.* developed a hybrid diagnostic system that combines a paper-based assay with an organic field-effect transistor to amplify the electrochemical signals generated by bacterial metabolic activity [47].

### Photonic readout techniques

Interactions between fast light and solid matter make photonic readout well-suited for novel and rapid AST. *Tanner et al.* summarized newly emerging phenotypic methods that use photonic or spectroscopic readouts [30]. For example, the BacterioScan^®^ system (BacterioScan Inc, Maryland Heights, Missouri, USA) is an FDA-cleared device for the detection of UTIs based on optical density and forward scatter intensity of a laser light source through a sample. Although its light scattering-based analyses have reached diagnostic sensitivities over 99%, it does not yet provide any information about the AMR profile of the pathogens involved [30, 48].

#### Vibrational spectroscopy

generates a spectrum that reflects the overall chemical composition of organic molecules, a process known as spectroscopic fingerprinting. These methods are label-free, allowing for simple sample preparation and cost-effective analysis [30, 49]. Furthermore, there is limited biomass required for recording the spectra avoiding time-consuming lab-cultures [50]. Two widely used vibrational techniques are infrared (IR) absorption spectroscopy and Raman spectroscopy.

#### IR spectroscopy

measures transitions between vibrational energy levels of organic molecules, producing an absorption spectrum [30]. Combined with Fourier transform (FT-IR) it allows for single cell analysis [49] and it is useful for accurate detection of bacterial variants [51]. Although primarily used for species identification [51], it is occasionally applied in AST. One major limitation, especially for POCT, is the strong background signal caused by water in aqueous samples [30].

#### Through raman spectroscopy

detects drug-induced biochemical changes using multivariate statistical analysis [52, 53]. AST results can be obtained after a few hours providing a decrease in TAT. For future clinical applications, determining bacterial resistance levels must be automated. Raman-based AST can be used both qualitatively and quantitatively. Sample preparation is straightforward and does not require staining or labeling; however, bacterial enrichment and preparation are typically still necessary at this stage. To improve analytical sensitivity, which is limited due to small Raman scattering cross-sections [49], surface-enhanced Raman spectroscopy (SERS) and stimulated Raman spectroscopy (SRS) have been developed [4, 30, 50]. *Samek et al.* reviewed the potential of SERS-based assays in POCTs as a reliable technique with a detection limit of 1 CFU/mL. Still, specialized instrumentation must be designed and developed [54].

*Zhang et al.*. developed a SRS-based assay to rapidly generate AST results using incorporation of deuterium oxide. The assay can detect antibiotic responses in as little as 10 min. Single-cell metabolism inactivation concentrations—translatable to MIC values—can be determined in under 2.5 h. However, clinical testing data is not available and the systems only allows detection for bacterial concentrations between 10^5^ and 10^6^ CFU/mL, necessitating pre-cultivation [4].

#### Scattered light integrated collection (SLIC)

combines laser light scattering, locked signal and integrating detection space to detect microorganisms at low concentrations. It can follow the growth of the microorganisms in real-time and evaluates the impact of different stresses on their growth and multiplication dynamics. A limit of detection of 10^2^ CFU/mL was found for commonly encountered pathogens in UTIs [55]. In a proof-of-concept, prospective clinical study, positive blood cultures flagged Gram-negative were tested with SLIC and compared with standard of care. A total of 505 bacterial-antimicrobial combinations were used to determine the categorical agreement to be 95.5% [56]. Another recent study showed that SLIC can be used to rapidly provide information on the identity of infecting microbes in musculoskeletal infections [57]. The major challenge for the implementation of SLIC in microbiological laboratories is the significant time attributed to culturing the infecting microbe prior to detection of its AMR profile [55–57].

#### Fluorescence-based

assays have been widely developed for bacterial infection diagnosis. These methods require fluorescent labeling of pathogens, allowing for the detection of diverse parameters depending on the type of fluorescent markers used, or the incorporation of a fluorescent/colorimetric dye into the medium that responds to the chemical environment allowing for real-time monitoring of the metabolic activity of bacteria.

#### Flow cytometry (FC)

enables single-cell analysis of bacteria by combining multiple fluorescence channels with forward and side scatter measurements providing information on the morphology and size of the particle [30]. FC can be performed directly on clinical specimens providing an early AMR profile. Moreover, the high throughput enables rapid screening of positive urine samples, reducing the number of samples that need to be analyzed in a microbiology laboratory [58]. A key challenge of FC remains the need to collect a sufficient number of events, which typically requires either large sample volumes or high bacterial concentrations (10^5^ CFU/mL) [30]. While FC holds clear potential for rapid AST, clinical research in this area remains limited [14].

*Velican et al.* studied the potential of FC for preliminary AST directly on urine specimens using artificial samples containing *E. coli* strains. A fluorescent marker indicating alterations in membrane potential was used and the obtained results were compared with culture methods. Data analysis revealed that AMR profiles could be generated within 4 h and there was good differentiation between resistant and susceptible pathogens [58].

FASTinov, a spin-off from the University of Porto (Portugal), has developed FAST kits that use panels of key antibiotics effective against both Gram-negative and Gram-positive bacteria. These kits can be applied directly to positive blood cultures and some are capable of identifying AMR mechanisms [59]. In a study by *Silva-Dias et al.*, 447 positive blood cultures were tested and the FASTgramneg and FASTgrampos kits showed categorical agreement rates of 96.8% and 98.6%, respectively, when compared to EUCAST standards. Results are available in under 2 h, significantly reducing TAT. However, pre-culturing is still required, and the pre-analytical steps must be performed by trained personnel [59].

#### Fluorescent/ colorimetric dyes

can also be added to the matrix in which the pathogens are grown allowing for monitoring the metabolic activity. In a proof-of-concept study by *Needs et al.*., microcapillary AST (mcAST) was applied directly to 12 clinical samples. The mcAST-POCT utilizes minimal equipment, monitoring bacterial growth via colirometric changes based on resazurin using a digital camera [29, 60]. The test demonstrated 100% accuracy in identifying urinary pathogens and achieved a 95% categorical agreement with standard AST. The device enables multiplexing, which allows for an expanded antibiotic panel or full MIC profiling. Results were available within 6 h, providing same-day results. However, it is debatable whether this meets the criteria of fast diagnostics for POCT. One of the major challenges of this system remains the unknown inoculum density influencing the time needed to obtain results [60].

Among the microfluidic methods, some use droplets to encapsulate bacteria within small volumes. This fine compartment enables detection of small changes in bacterial metabolism, multiplication dynamics and/or morphology [29, 61–63]. For example, *Avesar et al.* developed a platform based on stationary nanoliter droplet arrays combined with fluorescent detection of bacteria using resazurin. Ten bacteria-antibiotic combinations direct from clinical urine samples showed similar AST results as those obtained with routine practice [29]. Further improvements should be made to evolve the system into a genuine POCT device.

*Wat et al.* developed a microfluidic platform called SDFAST (Self Dilution for Faster Antimicrobial Susceptibility Testing), designed to streamline the mixing and dilution of bacterial suspensions with antibiotics. This two-layer chip enables MIC determination using a colorimetric WST-8 (water-soluble tetrazolium salt-8) readout. When tested on 51 clinical isolates, the system delivered results within 4 to 6 h, achieving over 90% essential agreement with standard gradient tests. Images are captured using a simple camera, and when combined with image analysis software and deep learning, the system shows strong potential for POCT [64].

*Aurchery et al.* developed a paper strip embedded masking tape (PASMAT) device that allows non-experts to perform length-based colorimetric AST. Five clinical isolates in combination with fifteen antibiotics were tested and MICs were determined and compared with BMD resulting in 100% similarity. Results must be confirmed in a clinical setting on a larger cohort and the manual procedures must be automated [10].

Microfluidic techniques have many benefits for developing portable devices, as they are easily handled and provide rapid results. However, their robustness is questioned. An important factor in deciding which microfluidic strategy to use, is the type of infection being targeted, the bacterial density in clinical specimens (potentially requiring additional culture steps) and the clinical pathway it is expected to add to or disrupt [27]. Compared to microfluidic devices, paper-based POCT devices overcome the challenges of high costs, material accessibility, and complex-replica-molding processes [10]. Besides, paper-based assays do not require an external power supply or excitation light source that electrochemical and fluorescent biosensors need [65]. Photonic assays hold significant promise for delivering low-cost diagnostic solutions, especially when labeling steps can be avoided [30]. The elimination of preculturing, but also the costs per assay and the clinical impact of the POCT device will play a crucial role in determining whether these methods gain acceptance among physicians and microbiologists. It remains to be seen which of the current approaches will prove most promising for further development.

## Genotypic AST

Most genotypic AST methods rely on nucleic acid amplification technologies (NAAT) to employ sensitive and specific detection of AMR genes. Common techniques are polymerase chain reaction (PCR), isothermal amplification and DNA micro-array testing [14]. The different commercially available rapid genotypic AST tests have been reviewed recently [13]. Most of these assays are PCR based and focus on specific organisms with specified resistance mechanisms. Some can be performed directly from specimen (blood cultures, swabs e.g.), reducing the TAT and approaching POCT, though some need purification [13].

The genotypic systems closest to POCT are the GeneXpert Family (Cepheid, CA, USA) and the BioFire^®^ (BioMérieux, France). Both systems are based on fully automated real time (reverse transcriptase) PCR combining sample preparation, nucleic acid extraction, amplification and detection of target sequences in one cartridge [66–68]. Similarly, Abbott (USA) developed a bench-top molecular system for qualitative detection of infectious diseases using isothermal amplification named ID NOW™ [69].

Since providing a complete overview of resistance genes is challenging, various kits have been developed over the years to detect single or multiple resistance markers. For example, the Xpert^®^ MRSA NxG kit by GeneXpert can detect methicillin-resistant *Staphylococcus aureus* (MRSA) directly from nasal swabs [70] and the ESBL-*ampC* assay allows for fast detection of ESBL (extended-spectrum beta-lactamase) genes [71]. Additionally, enzymes involved in resistance mechanisms, such as CTX-M β-lactamases produced by *Enterobacterales*, can be identified [72]. The implementation of these very rapid tests in clinical laboratories offers opportunities for early optimization of antibiotic treatment in patients. However, the relatively low positive predictive value increases the risk of carbapenem abuse [72, 73].

### Next-generation sequencing

Next-generation sequencing (NGS) has the theoretical potential to greatly impact on routine clinical microbiological diagnostics as it can be used to detect bacteria and to define epidemiological relatedness among bacterial isolates and to provide information on AMR genes [74]. The equipment needed is sophisticated, but its results can be directly applied in clinical settings [75]. NGS can sequence thousands of genes or entire genomes in a short time [14]. Two types of NGS are short-read and long-read sequencing provided by Illumina (San Diego, USA) and Oxford Nanopore Technologies (ONT, Oxford, UK) and can be performed on DNA of bacterial strains or all bacterial DNA present in clinical specimens [76].

Although NGS is extensively used in research studies, little focus is on the development and application in the microbiological laboratories due to the issues with standardization [7, 75–77]. Pre-analytical methods to reduce host DNA are needed to improve sequencing efficiency, though some host DNA can still offer insights into disease susceptibility [76]. Genotypic data will always need to be linked to phenotypic reference data. More research and refinement are needed to accurately link the composition of a resistome with the corresponding MICs [77, 78].

### CRISPR-Cas systems

CRISPR/Cas-systems are part of the adaptive immune defense mechanisms in prokaryotic cells. The RNA-guided system identifies and neutralizes invasive genetic elements by the specific binding of CRISPR RNA (crRNA) or guided RNA (gRNA) to the target sequencing triggering Cas enzymes to cleave both target-specific and nonspecific sequences [79]. Over the past few decades it has been used for gene modifications, but it could also be employed for resistance gene detection [14, 79, 80].

The very specific, RNA-guided binding of Cas proteins to their target has been combined with isothermal amplification by *Qin et al.* [44]. The target genes were cleaved in two short fragments and amplified using isothermal amplification which enabled direct detection of the resistance genes [14]. *Van de Veer et al.* developed LUNAS (Luminescent Nucleic Acid Sensor) employing CRISPR-Cas9 and a split luciferase to highly specifically detect nucleic acids. Combining the LUNAS sensor with an isothermal amplification steps allows detection of target at a load of 200 copies/µL in a single reaction mixture making it an attractive approach for POCT [81].

CRISPR-Cas-based biosensors are sensitive, specific and reliable [79] although their clinical application remains largely unexplored [14, 79]. The levels of automation of these assays is still limited. Besides, the crRNA and gRNAs used confine the targets implying that detecting drug-resistance-related mutations beyond these regions might be missed [79].

A key limitation of genotypic AST is its dependence on a predefined set of molecular targets, meaning its accuracy is constrained by current knowledge of resistance mechanisms and availability of corresponding genetic markers. In theory, it is possible to create a comprehensive catalog of all relevant genes within a single bacterial isolate. However, this process is highly labor-intensive and costly, and requires regular updating and quality control, making it impractical [7, 77]. Furthermore, while these methods can identify whether a resistance gene is present, they cannot confirm if the gene is actively expressed, specify the MIC or distinguish between live and dead organisms. Additionally, genotypic approaches are less reliable in detecting polymicrobial infections and often struggle to determine which specific organism carries the detected resistance genes [13–15, 77].

## Future perspectives

The laboratory landscape is evolving due to financial pressures from healthcare insurers, technological advancements, and the involvement of private investors [82]. This shift, along with the ongoing centralization of microbiology laboratories, has contributed to prolonged diagnostic TATs. Given the rising threat of AMR, there is an urgent clinical need for faster, near-patient AST solutions. The development and implementation of POCT devices capable of delivering AMR results holds promise for improving patient outcomes, shortening hospital stays, reducing healthcare costs, and enhancing antimicrobial stewardship. However, widespread adoption remains challenging due to technical complexities, limitations in pathogen detection, high costs, and regulatory barriers [13].

This review aimed to provide an overview of POC AST, ranging from regulatory-approved devices to proof-of-concept developments. Among these, only one approved POCT device—the PA-100—was identified. Especially in secondary health-care settings, where the PA-100 has a lower diagnostic accuracy, a novel or improved POCT device may have greater clinical impact. Future developments should aim to make the antibiotic panel more flexible, while increasing the range of detectable pathogens. Additionally, the devices must offer high throughput to handle the large volume of samples.

Numerous research groups are currently exploring novel phenotypic approaches, many of which show promise for future POCT device development. To enable direct-from-specimen testing, efforts must focus on improving sensitivity and specificity. Furthermore, strategies are needed to generate quantitative results that align with clinically relevant MIC values.

Genotypic AST methods are rapidly advancing. Emerging techniques, particularly NGS, now enable comprehensive analysis of the microbiome and resistome within clinical samples. Although these methods rely on prior knowledge of resistance genes, they offer the potential to transform microbiology laboratories. Future work should prioritize cost reduction in genotypic methods, streamline equipment design, increase knowledge about resistance mechanisms, and enhance strategies for accurate phenotypic resistance prediction.

The introduction of POCT devices is also reshaping the economic landscape of clinical diagnostics. These devices reduce the need for hands-on time by specialized personnel and enable immediate treatment adjustments, potentially shortening hospital stays. Furthermore, they decrease the number of specimens processed in central microbiological laboratories and reduce the physical space required for diagnostic equipment.

Importantly, the use of POCT devices requires well-defined roles and responsibilities. Each POCT device comes with manufacturer-specific instructions regarding quality control and specimen handling. Besides, the devices should be interfaced with patient electronic medical records, preventing manually entering of results which may lead to errors, delays and duplications and software is needed to collect all the data to check congruency between instruments [83]. On the other hand, development of new tests will not replace the existing methods anytime soon. Complementing classical tests with POCT will lead to a broader range of diagnostic tools, each suited to different diseases or clinical settings. As a result, the overall workflow becomes more complex and standardization becomes harder.

In the distant future, the expansion of POCT may necessitate a reorganization of laboratory services, with POCT performed at or near the bedside, supported by hospital‑based laboratories for urgent, first‑line analyses and centralized core laboratories dedicated to high‑throughput and specialized testing without TAT constraints.

The recent surge in artificial intelligence (AI) presents new opportunities for its integration into POCT given its ability to process vast volumes of data. Combining machine learning with AI can provide clinical decision support for physicians and workflow improvements for hospitals [84]. As technological advancements continue and economies of scale are realized, these innovations are expected to become increasingly accessible and affordable.This evolution may also prompt important discussions around responsibility, accountability, and the ethical implications of AI-driven diagnostics [16].

Although POCT may demonstrate high sensitivity and specificity in detecting infectious agents, its clinical impact remains a subject of debate. During the literature search for this review, substantial diagnostic performance data were identified. However, clinical data, evidence regarding the influence of POCT devices on clinical decision-making, particularly in terms of antibiotic prescribing behavior, is limited. While some studies report a (moderate) reduction in inappropriate antibiotic prescriptions following POCT implementation [85, 86] and some state the opposite [87], comprehensive data on its broader impact on clinical workflows and therapeutic choices still need to be generated.

## Data Availability

No datasets were generated or analysed during the current study.
